# Long Non-Coding RNA H19 Promotes Glioma Cell Invasion by Deriving miR-675

**DOI:** 10.1371/journal.pone.0086295

**Published:** 2014-01-23

**Authors:** Yan Shi, Yingyi Wang, Wenkang Luan, Ping Wang, Tao Tao, Junxia Zhang, Jin Qian, Ning Liu, Yongping You

**Affiliations:** 1 Department of Neurosurgery, The First Affiliated Hospital of Nanjing Medical University, Nanjing, China; 2 Liver Transplantation Center, The First Affiliated Hospital of Nanjing Medical University, Nanjing, China; 3 Surgical Research Center, Medical School, Southeast University, Nanjing, China; 4 Department of Neurosurgery, People's hospital of Xuancheng city, Anhui, China; Beijing Tiantan Hospital, Capital Medical University, China

## Abstract

*H19* RNA has been characterized as an oncogenic long non-coding RNA (lncRNA) in breast and colon cancer. However, the role and function of lncRNA H19 in glioma development remain unclear. In this study, we identified that H19/miR-675 signaling was critical for glioma progression. By analyzing glioma gene expression data sets, we found increased H19 in high grade gliomas. H19 depletion via siRNA inhibited invasion in glioma cells. Further, we found H19 positively correlated with its derivate miR-675 expression and reduction of H19 inhibited miR-675 expression. Bioinformatics and luciferase reporter assays showed that miR-675 modulated Cadherin 13 expression by directly targeting the binding site within the 3′ UTR. Finally, introduction of miR-675 abrogated H19 knockdown-induced cell invasion inhibition in glioma cells. To our knowledge, it is first time to demonstrate that H19 regulates glioma development by deriving miR-675 and provide important clues for understanding the key roles of lncRNA-miRNA functional network in glioma.

## Introduction

It has been reported that about 98% of the “junk” DNAs are transcribed as non-coding RNAs (ncRNAs) including short ncRNAs (which include, but are not limited to, microRNAs) and long ncRNAs (lncRNAs) [1]. LncRNAs, are defined as endogenous cellular RNAs of more than 200 nucleotides in length and implicated in a myriad of molecular functions, such as modulation of alternative splicing, chromatin remodeling and RNA metabolism [2], [3], [4], [5]. Although lncRNAs are longer than, and functionally as well as structurally distinct from known endogenous small RNAs such as microRNAs, there are some connections between these RNA classes: a small number of lncRNA genes harbor internally encoded small RNAs and certain lncRNAs may acquire functionality by acting as the precursor to small RNAs capable of regulatory function, such as microRNAs[6]. To date, emerging evidence has strongly suggested that aberrant microRNA expression is a feature of human glioblastoma[3]. However, lncRNA-miRNA network in glioma remains unknown and needs further investigation.

LncRNA H19 is produced from imprinted genes *H19*, whose expression is depending on the parental origin of the chromosome. LncRNA H19 has been considered as an oncogenic lncRNA in hepatocellular and bladder carcinoma [7], [8], [9], [10], [11]. Matouk et al. found that H19 is significantly elevated after exposure to hypoxia, and H19 has pro-tumorigenic properties [12]. Berteaux et al. demonstrated that lncRNA H19 is actively linked to E2F1 (E2F transcription factor 1) to promote cell-cycle progression of breast cancer cells [13]. But the underlying role and mechanism of H19 involved in glioma development remains unclear.

In the current study, we explored the clinical feature, biological function and potential mechanism of lncRNA H19 in glioma. We found that H19 was closely correlated with tumor grade in 3 different glioma data sets. Moreover, as a precursor, H19 derived miR-675 and then regulated Cadherin 13 (CDH13) which is the directly target of miR-675, thereby modulating glioma cell invasion. The oncogenic function of H19/miR-675 signaling may serve as the potential target for glioma therapy.

## Materials and Methods

### Human tissue samples and cell lines

158 glioma data with mRNA and miRNA expression microarray were downloaded from Chinese Glioma Genome Atlas (CGGA) data portal (http://www.cgga.org.cn/portal.php). The samples comprised 48 astrocytomas (A, WHO Grade II), 13 oligodendrogliomas (O, WHO Grade II), 8 anaplastic astrocytomas (AA, WHO Grade III), 10 anaplastic oligodendrogliomas (AO, WHO Grade III), 15 anaplastic oligoastrocytomas (AOA, WHO Grade III) and 64 GBM (WHO Grade IV). High grade glioma (HGG) including AA, AO, AOA and GBM. Low grade glioma (LGG) including O and A. Glioma gene expression data sets are deposited at Rembrandt data (https://caintegrator.nci.nih.gov/rembrandt/) and the Gene Expression Omnibus Web site (http://www.ncbi.nlm.nih.gov/geo/, accession No. GSE16011). The human U87 and U251 glioblastoma cell lines were purchased from the Chinese Academy of Sciences Cell Bank. The cells were grown in Dulbecco's modified Eagle's medium (DMEM) (Gibco,Los Angeles, CA, USA),supplemented with 10% fetal bovine serum in an atmosphere containing 5% CO_2_ at 37°C.

### Oligonucleotides and transfection

The 2′-O-methy1 (2′ – OMe-) oligonucleotides were chemically synthesized by GenePharma (Shanghai, China). The sequences are: H19 small interfering RNA (siRNA) 1: sense, 5′-CCC ACA ACA UGA AAG AAA CTT-3′, antisense: 5′-AUU UCU UUC AUG UUG UGG GTT-3′; H19 small interfering RNA (siRNA) 2: sense, 5′-GCU AGA GGA ACC AGA CCU UTT-3′, antisense: 5′-AAG GUC UGG UUC CUC UAG CTT-3′; A siRNA that was unrelated to any human sequence was used as a negative control (NC): sense, 5′-UUC UCC GAA CGU GUC ACG UTT-3′, antisense: 5′-ACG UGA CAC GUU CGG AGA ATT-3′; 2′-OMe-hsa-miR-675 inhibitor: 5′-UGA GCG GUG AGG GCA UAC AG-3′; 2′-OMe-hsa-miR-675 mimics: sense, 5′-CUG UAU GCC CUC ACC GCU CA-3′, antisense: 5′-AGC GGU GAG GGC AUA CAG UU-3′; MircoRNA inhibitor negative control(NC), 5′-CAG UAC UUU UGU GUA GUA CAA-3′. Oligonucleotides (20 µM) were transfected into U87 and U251 glioblastoma cells using Lipofectamine 2000 (Invitrogen) following the manufacturer's instructions.

### RNA extraction and Quantitative RT-PCR

RNA was extracted from cells after transfected using TRIzol (Invitrogen) following the manufacturer's protocol. To detect the levels of miR-675 in cells, reverse transcription (RT) was conducted with the Applied Biosystems® TaqMan® MicroRNA Reverse Transcription Kit (Applied Biosystems, Foster City, CA). The primers for the miR-675 were purchased from Guangzhou Ribo BioCoLTD (Guangzhou, China). U6 was used for normalization. The ABI StepOne Plus (Applied Biosystems, Foster City, CA) was used to perform the amplification reaction. And the date was analyzed by the 2^−ΔΔCt^ method. The PCR reaction for

H19 was performed as previously described [13], [14]. Each experiment was performed in triplicate.

### In vitro invasion assay

Cell invasion was determined by the transwell assay and the scratch wound assay. Transwell assay: the related oligonucleotides were transfected into the cells according to the protocol. After incubated for 48 hours, 3×10^4^ cells were transferred on the top of the Matrigel-coated invasion chambers (BD Biosciences, San Jose, USA) in a serum-free DMEM and we add the DMEM containing 10% fetal bovine serum to the lower chamber. After 24 h, non-invasion cells were removed, and the invading cells were fixed with 95% ethanol, stained with0.1% crystal violet, photographed (×100). Tests were repeated via three independent experiments. Scratch wound assay: the related oligonucleotides were transfected into the cells in six-well plates. Cell layers were scratched using a sterile pipette tip to form wound gaps. The wound location in the six-well plates was marked. Cells were photographed to record the wound width (0 h). Twelve (U87) or twenty-four hours later (U251), photographs will be taken again at the marked wound location to measure the cell migration ability.

### Luciferase assay

The 3′-UTR of CDH13 containing the putative miR-675 binding sequences was cloned into a firefly luciferase reporter construct. The 3′-UTR of CDH13 without the putative miR-675 bindng sequences was used as mutated controls (Invitrogen). The pGL3-WT-CDH13-3′UTR-Luc/pGL3-MUT-CDH13-3′UTR-Luc and miR-675 mimics/miR-675 inhibitor were co-transfection into the cells. Luciferase activity was measured using the Dual-Luciferase Reporter Assay System (Promega, USA).

### Western blotting

The oligonucleotides were transfected into the cells. Proteins were extracted from cells with RIPA lysis buffer (KenGEN, China) and were quantified using a BCA Protein Assay Kit (Beyotime, China). We add the 30 µg of protein lysates to SDS-PAGE. The electrophoresed proteins were transferred to PVDF membranes membranes (Millipore, USA). The membrane was blocked in 5% nonfat milk and incubated with diluted antibodies against CDH13 (1∶200; Santa Cruz; USA), followed by incubation with a horseradish peroxidase-conjugated secondary antibody (1∶2500; Santa Cruz; USA). Glyceraldehyde-3-phosphate dehydrogenase (GAPDH) was used as a control (1∶1000, CST, USA).

### Statistical analysis

All experiments were performed three times. All data are presented as mean ± standard error. Data were analyzed with SPSS 10.0.Statistical. Evaluation of the data was performed by t-test (two-sided) and one-way-ANOVA. P<0.05 was considered statistically significant.

## Results

### H19 expression correlates with glioma grade

**Figure 1:**
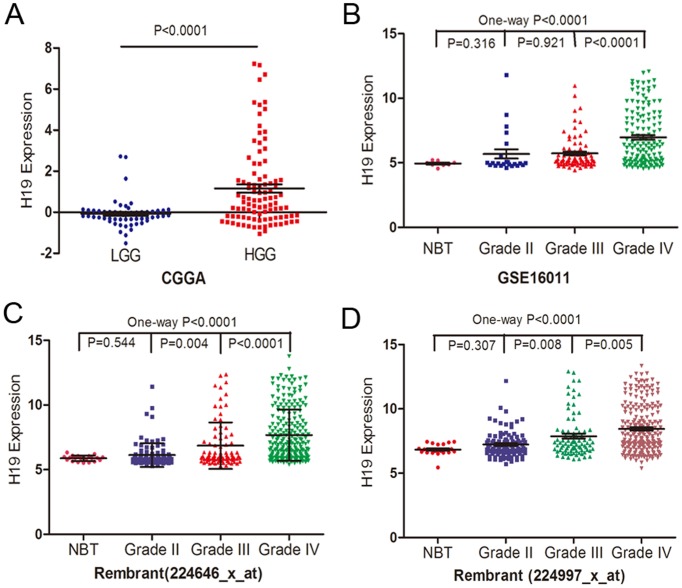
H19 expression in glioma tissues. (A) H19 levels were analyzed in glioma tissues of the CGGA glioma datasets (61 cases of grade II, 33 cases of grade III and 64 cases of grade IV). (B) The expression of H19 was analyzed in glioma tissues of the GSE16011 glioma datasets. (C–D) H19 expression with two probes was analyzed in glioma tissues of the Rembrant glioma datasets.

We initially analyzed H19 expression pattern in whole genome gene profiling of 158 glioma tissues in CGGA data and found that, as shown in Fig. 1A, H19 expression was significant higher in HGG tissues than in LGG ones (P<0.0001). Further, two independent glioma gene expression data sets (Rembrandt data and GSE16011 data) were employed to examine the association between H19 expression levels and glioma grade (Fig. 1 B–D). One-way ANOVA analysis showed that H19 expression levels were significantly associated with tumor grade (P<0.0001 for both Rembrandt data and GSE16011 data), which was similar with the CGGA data. These findings demonstrate that H19 may play an important role in glioma progress.

### H19 induced invasion of glioma cell

**Figure 2:**
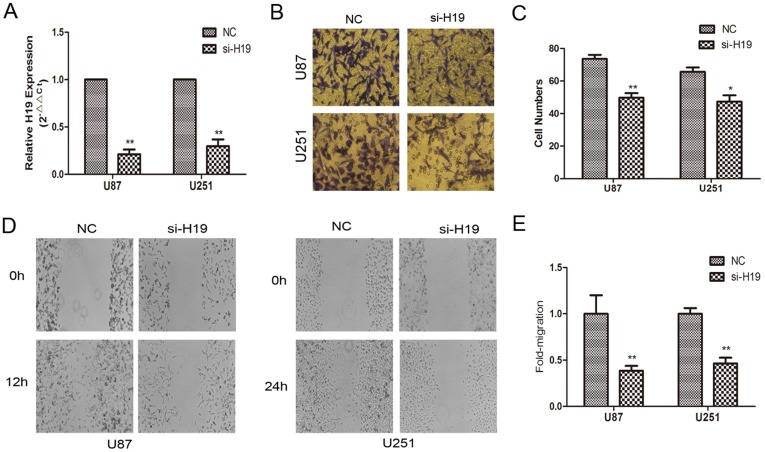
Evaluation of biological functions of H19 in U87 and U251 cells. (A) Transfection efficiency of si-H19 in U87 and U251 cells was indicated by PCR. (B–C) si-H19 inhibited the invasiveness of U87 and U251 cells. Cells were examined for cell invasion in 24-well plates with transwell chambers. Migrated cells were stained with crystal violet. The invasiveness of U87 and 251 cells was attenuated with the decreased expression of H19. (D–E) Wound healing assay of glioma cells transfected with either control or the si-H19, respectively. The wound healing was photographed at different time points and wounded gaps were analyzed by measuring the distance of migrating cells for 3 different areas for each wound. *P<0.05, **P<0.01.

To explore the effect of H19 RNA on cell invasion in glioma, H19 was down-regulated by si-RNA (Fig. 2 A). The effects of H19 RNA on the invasiveness and migration of glioma cells were checked by Transwell and wound healing assays. An *in vitro* Matrigel invasion assay revealed that invasiveness of U87 and U251 cells transfected with si-H19 were suppressed compared with negative control (Fig. 2 B and C). The results of *in vitro* wound healing assay displayed that the levels of H19 was decreased, the migration of U87 and U251 cells was significantly attenuated compared with the control cells (Fig. 2 D and E).

### H19 is a developmental reservoir of miR-675 in promote cell invasion

**Figure 3:**
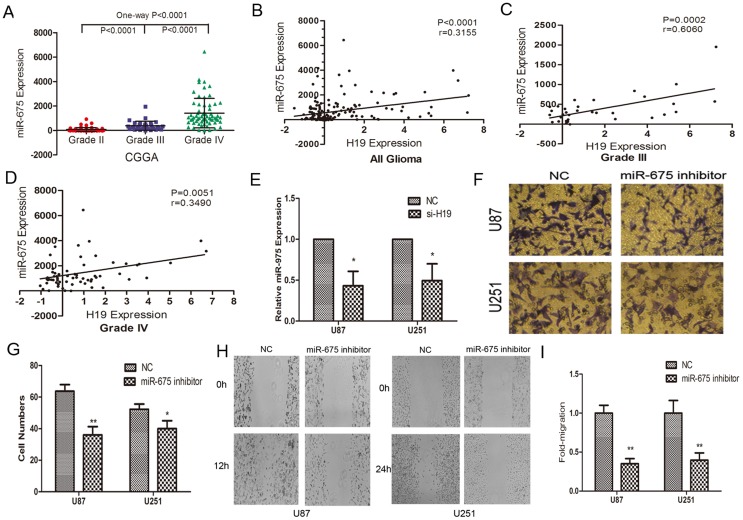
H19 regulates the expression of miR-675 which is associated with cell invasion. (A) MiR-675 expression in 158 glioma tissues of the CGGA glioma datasets. (B) The data shows that miR-675 positively correlated with H19, in 158 glioma samples. (C–D) MiR-675 positively correlated with H19 in HGG of 158 glioma samples. (E) si-H19 decreases miR-675 expression compared with NC in U87 and U251 cells. (F–G) The effects of miR-675 inhibitor on the invasion of U87 and U251 cells were assessed by transwell invasion assay. (H–I) The results of in vitro scratch wound assay showed that knockdown miR-675 inhibit the migration of glioma cells. *P<0.05, **P<0.01.

H19 is reported to be the primary precursor of two distinct miRNAs (miR-675-5p and miR-675-3p), in which transfection with H19 complementary DNA containing the pri-miR-675 hairpin increased the expression of mature miR-675 in human kidney 293T cells and it has been suggested that it may be these miRNAs that confer functionality on H19 [15], [16], [17]. Thus, we analyzed miR-675 expression level in 158 glioma tissues in CGGA data. One-way ANOVA analysis showed that miR-675 was significantly associated with tumor grade and miR-675 expression was significant higher in high grade glioma than in low grade glioma (Fig. 3 A). To evaluate the potential correlativity in aberrant expression of H19 and miR-675 expression values of 158 glioma specimens in CGGA data, Pearson correlation assay revealed a significant and positive correlation between H19 and miR-675 (Fig. 3 B–D). We then found that H19 knockdown remarkably reduced miR-675 expression in U87 and U251 cells (Fig. 3 E). Furthermore, we also found that miR-675 inhibition significantly suppressed cell invasion in U87 and U251 glioma cells (Fig. 3 F–I).

### Cadherin 13 is a direct target of miR-675

**Figure 4:**
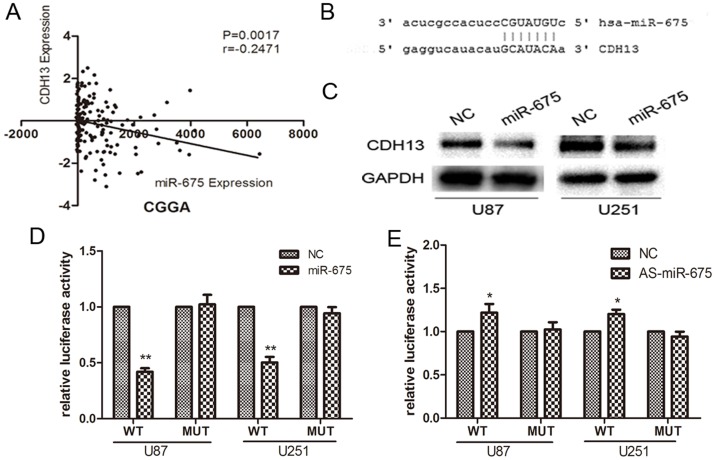
CDH13 is a directly target of miR-675. (A) Correlations of miR-675 with CDH13 in glioma tissues. (B) Putative binding sites of miR-675 within theCDH13 3′UTR, as predicted by miRanda and Pictar alghorithms. (C) CDH13 protein levels were measured in U87 and U251 cells at 48 h post-transfection. (D) MiR-675 down-regulated luciferase activities controlled by wild-type CDH13 3′UTR, but did not affect luciferase activity controlled by mutant CDH13 3′UTR. The luciferase activity was measured by dual-luciferase reporter assay (Promega) and was normalized to Renilla luciferase activity. *P<0.05, **P<0.01.

To investigate the mechanism of action of miR-675 in glioma cell invasion, we performed bioinformatic analysis, using miRanda and Pictar alghorithms, and found that the “seed sequence” of miR-675 matched the 3′ UTR of the CDH 13 gene (Fig 4A). We used Pearson correlations to analyze the relationship between miR-675 and CDH13 in 158 glioma specimens from CGGA date and determined the expression of miR-675 was correlated with CDH13 in glioma (P = 0.0017; Fig. 4B). Further, Western blot analysis showed that increasing miR-675 expression in cells led to a decrease in CDH13 protein (Fig. 4C). To indicate the direct interaction between miR-675 and its binding site within 3′ UTR of CDH13, we created pGL3-WT-CDH13-3′UTR plasmids and pGL3-MUT-CDH13-3′UTR. Luciferase reporter assays showed that over-expression of miR-675(supplementary Fig.S1) led to a marked decrease of luciferase activity of PGL3-WT-CDH13-3′UTR and knock-down miR-675(supplementary Fig. S2) led to an increase of the luciferase activity in U87 and U251 cells without change in luciferase activity of PGL3-MUT-CDH13-3′UTR (Fig. 4 D and E). These results indicate that miR-675 directly modulate CDH13 expression by binding 3′ UTR of CDH13.

### Expression of miR-675 overrides si-H19-induced modulation of invasion in glioma

**Figure 5:**
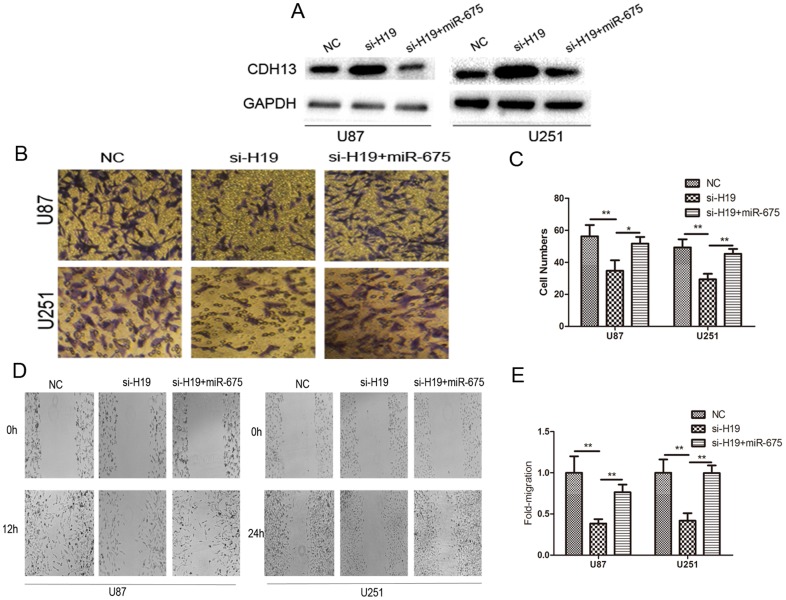
Effect of H19/miR-675 signaling on glioma cell biology. (A) CDH13 expression changes following transfection with si-H19 and miR-675 identified by Western blots. (B–C) Effect of si-H19 and miR-675 on glioma cell invasion, after differential treatment in the *in vitro* transwell invasion assay. (D–E) Cell migration change was analyzed by scratch wound assay. *P<0.05, **P<0.01.

Having demonstrated the relationship between lncRNA H19 and miR-675 by our present data, the importance of miR-675 in lncRNA H19-mediated cell invasion is still unclear in glioma cells. Western blot analysis showed that miR-675 largely abrogated the effect of si-H19 on elevating the expression of CDH13 (Figure 5 A). As shown in Fig. 5 B and C, increased miR-675 in lncRNA H19-depleted cells rescued the invasion phenotype induced by si-H19 transfection. Moreover, expression of miR-675 largely rescued the effect of si-H19 on cell migration (Fig. 5 D and E). Our results strongly indicated that miR-675 is a critical participant of H19 involved in cell invasion.

## Discussion

H19 is transcribed in an untranslated RNA molecule (lncRNA H19) [18] that lacks conserved open reading frames but does have a conserved secondary RNA structure [19], which accumulates in the human placenta and several fetal tissues, and probably plays a pivotal role in embryogenesis and fetal growth and development [20]. H19 is located on chromosome 11 p 15.5 and lies within 200 kbp downstream of the IGF-2 gene. These two genes are imprinted in opposite directions, so that the paternal IGF-2 and the maternal H19 alleles are selectively expressed [21], [22]. Extensive deletions and/or point mutations in the 5′-long untranslated region of an ectopic human H19 RNA enable 26-kDa protein translation, but no endogenous translation product has so far been identified[23], [24]. Therefore, it was proposed that H19 functions as a riboregulator [18]. Since the first mention of H19 in 1984 by Pachnis et al[25], its functions remain enigmatic. It has been suggested that H19 functions as a tumor suppressor in some Wilms' tumors, embryonic rhabdomyosarcoma, and the Beckwith-Wiedmann cancer predisposing syndrome [26], [27], [28]. Furthermore, ectopic expression of the H19 gene in human embryonic tumour cell lines leads to loss of clonogenicity and reduced tumourigenicity in nude mice[6], [29]. However, several other studies [29] have shown that H19 characterized as oncogenic factor in breast adenocarcinoma, bladder tumor and choriocarcinoma. Our study also demonstrated that H19 play a key role in contributing tumorigenesis. H19 was associated with tumor grade, and H19 repression inhibited glioma cell invasion.

Smits et al. [30] showed a high conservation of H19 over 148 Ma of mammalian evolution and suggested that the H19 transcript have a dual role: first as the full-length transcript based on the conservation of exon-intron structure and second as the miR-675 precursor based on the sequence similarity. Recently, H19 was reported to be the primary miRNA precursor of miR-675 in both human and mice [16]. In our in this study, we showed that both miR-675 and its precursor H19 expressions are elevated in HGG tissues compared with LGG ones. In addition, miR-675 was found to positively correlate with H19 expression in CGGA microarray data. *In vitro* experiment showed that deprivation of H19 expression remarkably reduced miR-675 expression in glioma cells. Such correlation is in agreement with the findings from the present study showing that H19 is the precursor of miR-675 in glioma. Furthermore, our results proved that the effect of miR-675 on invasion in glioma cells by directly targeting CDH13. Moreover, further study implies miR-675 largely abrogated the effect of si-H19 on elevating the invasion of glioma cells. In short, the present study confirmed the important role of the miR-675 pathway in the biological function of H19.

According to our data, the oncogenic function of H19/miR-675 is featured by targeting the nonclassical cadherin CDH13. In recent years, the associations of CDH13 with human cancers have been proposed. Current studies have highlighted the role of CDH13 as a tumor suppressor in lung cancer, breast cancer and malignant melanoma, and so on[31]. But the effect of CDH13 on glioma is still poorly understood. In the present study, we firstly showed the role of the H19/miR-675/CDH13 pathway in glioma development. However, more function of H19 besides provide miR-675 in glioma need further investigation.

In summary, H19 and its derivate miR-675 were positively correlated with glioma grade. And H19 regulated glioma cell invasion by deriving miR-675 and inhibited CDH13. Here, we find lncRNA directly regulates miRNA expression, and plays a “trigger” role in inducing invasion in glioma by deriving miRNA. To our knowledge, this is the first study to show the role and function of H19 in glioma. Therefore, understanding the key role of “lncRNA-miRNA” module in glioma will lead to the identification of new therapeutic targets for treating glioma and warrants further investigation.

## Supporting Information

Figure S1 **Transfection efficiency of miR-675 mimics in glioma cells.** The expression of miR-675 was up-regulated by miR-675 mimics in U87 and U251 cells and the levels of miR-675 were indicated by PCR. *P<0.05, **P<0.01.
(TIF)

Figure S2 **Transfection efficiency of AS-miR-675 in U87 and U251 cells.** MiR-675 was down-regulated by AS-miR-675 and the levels of miR-675 were indicated by PCR. *P<0.05, **P<0.01.
(TIF)
