# Supplementary figures and images for: Long Non-Coding RNA H19 Promotes Glioma Cell Invasion by Deriving miR-675
Source: PLoS One. 2014 Jan 23;9(1):e86295. doi: 10.1371/journal.pone.0086295 (PMC3900504; doi:10.1371/journal.pone.0086295)

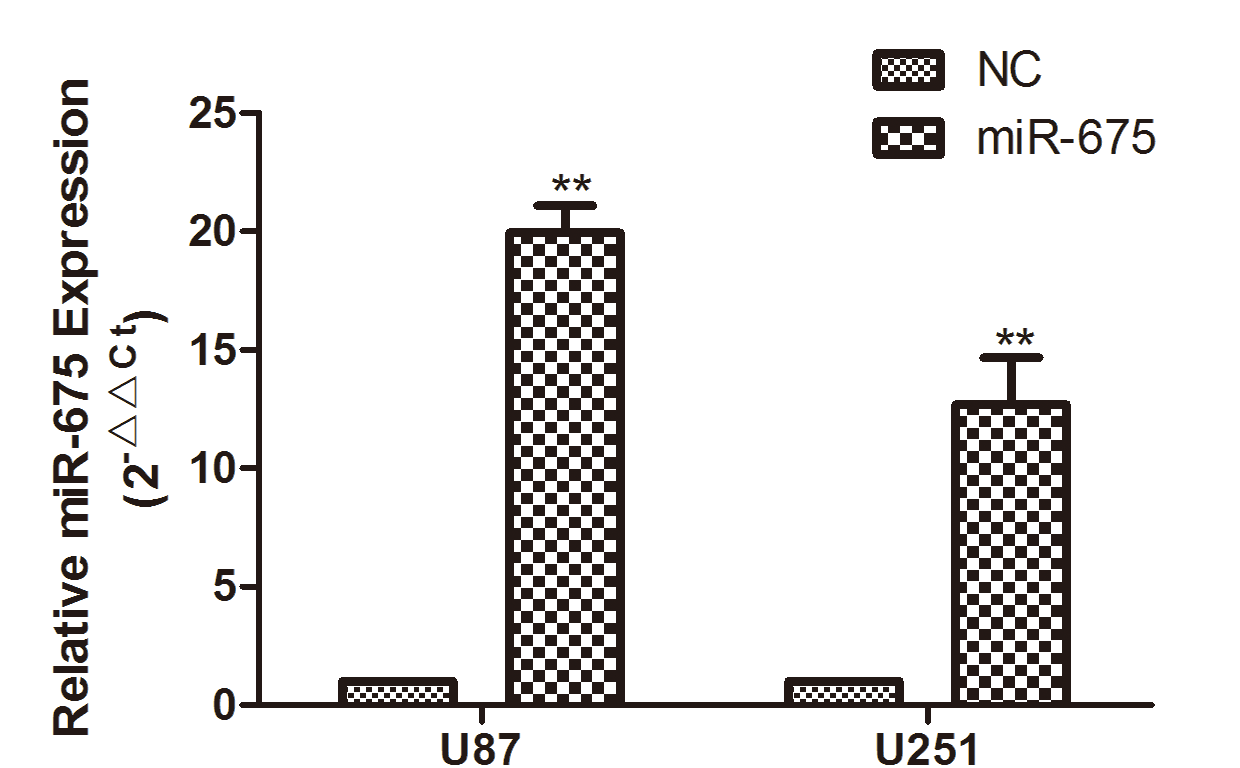

Supplement: Figure S1 — Transfection efficiency of miR-675 mimics in glioma cells. The expression of miR-675 was up-regulated by miR-675 mimics in U87 and U251 cells and the levels of miR-675 were indicated by PCR. *P<0.05, **P<0.01. (TIF) [file pone.0086295.s001.tif]

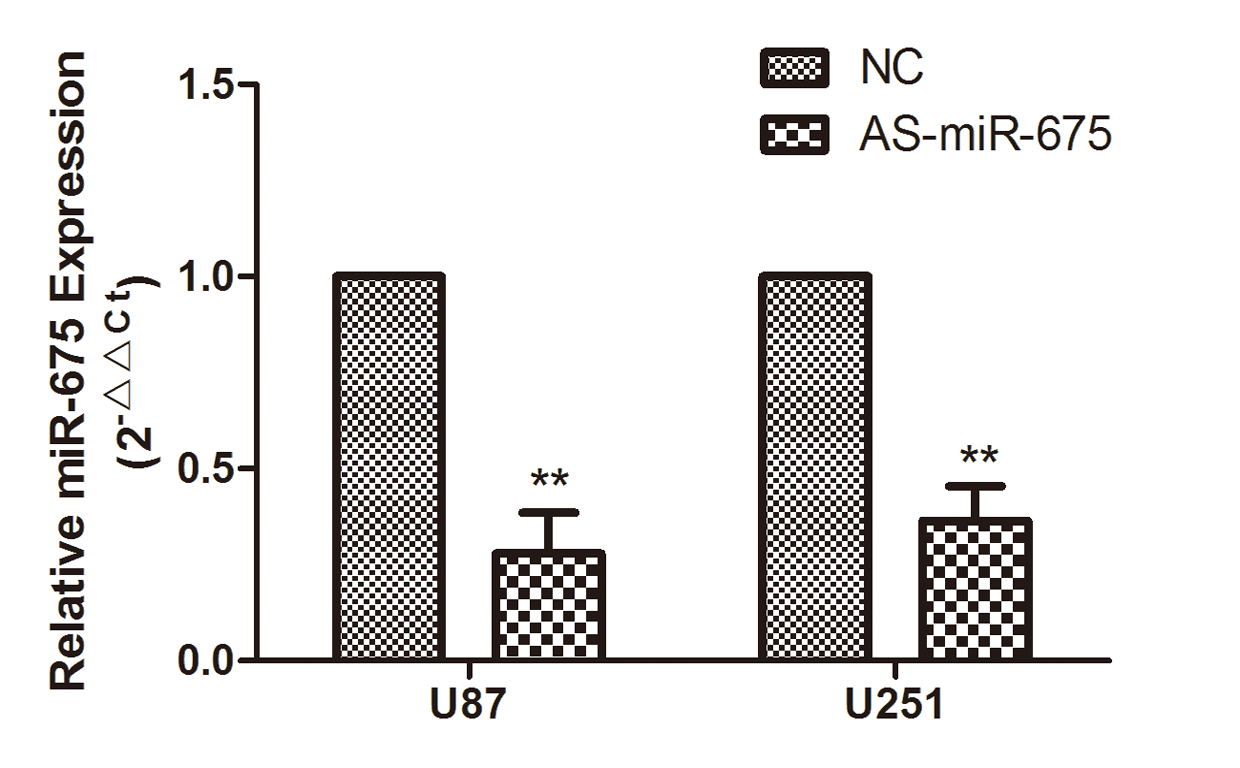

Supplement: Figure S2 — Transfection efficiency of AS-miR-675 in U87 and U251 cells. MiR-675 was down-regulated by AS-miR-675 and the levels of miR-675 were indicated by PCR. *P<0.05, **P<0.01. (TIF) [file pone.0086295.s002.tif]
